# Association Between Digital Isolation and Sleep Disorders in Older Adults: Cross-Sectional and Longitudinal Study Using National Health and Aging Trends Study (NHATS) Data

**DOI:** 10.2196/75328

**Published:** 2025-08-06

**Authors:** Shijun Yang, Wei Tan, Fan Yang

**Affiliations:** 1 Department of Cardiology, Union Hospital, Tongji Medical College, Huazhong University of Science and Technology, Wuhan, China; 2 Hubei Key Laboratory of Biological Targeted Therapy, Union Hospital, Tongji Medical College, Huazhong University of Science and Technology, Wuhan, China; 3 Hubei Provincial Engineering Research Center of Immunological Diagnosis and Therapy for Cardiovascular Diseases, Union Hospital, Tongji Medical College, Huazhong University of Science and Technology, Wuhan, China; 4 Geriatric Hospital Affiliated With Wuhan University of Science and Technology, Wuhan, China; 5 Department of Radiology, Union Hospital, Tongji Medical College, Huazhong University of Science and Technology, Jiefang Avenue #1277, Wuhan, 4300, China, 86 13871266365; 6 Precision Radiology & Interventional Medicine, Hubei Provincial Clinical Research Center, Union Hospital, Tongji Medical College, Huazhong University of Science and Technology, Wuhan, China; 7 Hubei Key Laboratory of Molecular Imaging, Union Hospital, Tongji Medical College, Huazhong University of Science and Technology, Wuhan, China

**Keywords:** digital isolation, sleep disorders, older adults, cross-sectional study, longitudinal study, mental health, mobile phone

## Abstract

**Background:**

As digital technology becomes increasingly embedded in daily life, digital isolation among older adults has become more pronounced. This isolation may restrict access to health information and social support, potentially leading to poorer sleep quality. However, most existing studies on digital isolation and sleep disorders were cross-sectional, lacking longitudinal evidence to establish causality.

**Objective:**

This study aims to investigate the association between digital isolation and sleep disorders in older adults using both cross-sectional and longitudinal designs and to assess the impact of specific components of digital isolation on the risk of sleep disorders.

**Methods:**

We analyzed data from the National Health and Aging Trends Study (NHATS) collected from 2011 to 2022, including a discovery sample of 5989 older adults and a validation sample of 3443. Digital isolation was measured by the use of mobile phones, computers, email, and the internet, while sleep disorders were identified based on difficulties initiating or maintaining sleep and the use of sleep medication. Multivariable logistic regression and Cox proportional hazards models were used for cross-sectional and longitudinal analyses, respectively.

**Results:**

Cross-sectional analyses revealed a higher prevalence of sleep disorders among those with high digital isolation (discovery: 1452/2166, 67.03% vs 2259/3823, 59.06%; odds ratio [OR] 1.23, 95% CI 1.09-1.39; *P*<.001 and validation: 673/960, 70.10% vs 1524/2483, 61.38%; OR 1.22, 95% CI 1.02-1.47; *P*=.03). In longitudinal analyses, high digital isolation was associated with an increased risk of sleep disorders in the discovery (hazard ratio [HR] 1.21, 95% CI 1.05-1.38; *P*=.006) and pooled samples (HR 1.17, 95% CI 1.05-1.31; *P*=.005), but the association was not statistically significant in the validation sample after adjustment (HR 1.11, 95% CI 0.91-1.36; *P*=.30).

**Conclusions:**

Digital isolation is significantly associated with sleep disorders among older adults, particularly in cross-sectional analyses, while longitudinal findings provide partial support for this association. The nonsignificant result observed in the validation sample may reflect sample heterogeneity and suggests that mental health may mediate this relationship. Future interventions should address mental health to help mitigate the negative impact of digital isolation on sleep.

## Introduction

Technology has advanced significantly and is now integrated into nearly every aspect of daily life, including information retrieval, social communication, and entertainment [1]. Devices such as smartphones, the internet, and computers have greatly facilitated social connectivity. However, not everyone has embraced this digital transformation. A significant portion of the population, particularly older adults, remains excluded—a phenomenon referred to as “digital isolation” [2-4]. Digital isolation refers to the inability or unwillingness to use digital technologies such as the internet or electronic devices, often due to lack of access, skills, or interest [5]. This issue is particularly pronounced among older adults. Barriers include financial constraints, limited digital literacy, or lack of previous exposure to such technologies. In some cases, physical or cognitive health issues—such as motor impairments or memory decline—further hinder technology adoption [6]. The implications of this digital divide extend beyond mere entertainment. It can restrict access to essential information and services, increase social isolation, and negatively impact mental health. Studies indicate that digitally isolated older adults are more prone to loneliness, which can negatively impact mental health, potentially contributing to conditions like depression and anxiety [7-12].

Sleep disturbances are notably prevalent among older adults and tend to increase with age, with more than half reporting difficulties initiating or maintaining sleep, or experiencing early morning awakenings [13,14]. Digital isolation, as described above, may be a significant contributing factor to these sleep disturbances. This connection can operate through several mechanisms; for instance, limited internet access may reduce exposure to beneficial sleep hygiene information or behavioral recommendations [15-19], while various digital tools that could enhance sleep quality—such as meditation apps or sleep tracking technologies—are less likely to be used by digitally isolated individuals [15,20]. Furthermore, by hindering access to digital technologies that facilitate social connections (eg, video calls with family), digital isolation can exacerbate loneliness and related mental health issues like depression and anxiety [7-12], which are established risk factors for poor sleep. Given that poor sleep is associated with serious health outcomes, including cardiovascular disease, diabetes, cognitive decline, and impaired immune function [17-19,21,22], identifying determinants like digital isolation is crucial for promoting healthy aging. However, while much research has focused on the negative impacts of excessive technology use in younger populations [23-25], the effects of digital lack and isolation on sleep in older adults remain understudied, particularly concerning the establishment of causal relationships [26]. Existing studies have been primarily cross-sectional, providing only a snapshot at a single time point, making it difficult to ascertain causality. In summary, digital isolation may affect sleep quality through multiple pathways as described above, but the causal direction of this association and specific mechanisms still require further verification.

Therefore, to address these research gaps, this study aims to evaluate the cross-sectional association between the degree of digital isolation and the prevalence of sleep disorders in older adults and to assess whether digital isolation increases the risk of developing sleep disorders in the future through longitudinal analysis. In addition, we will analyze the effects of different components of digital isolation (eg, use of phone, computer, email, and the internet) on the risk of sleep disorders. We hypothesize that higher levels of digital isolation will be associated with an increased risk of sleep disorders among older adults.

## Methods

### Study Population

We analyzed data from the National Health and Aging Trends Study (NHATS), which has been collecting data since Wave 1 in 2011. For the discovery sample, we used data from Wave 1 (2011) through Wave 8 (2018), encompassing a total of 7 waves of follow-up. In Wave 1, 8245 participants were initially recruited into the study. To ensure analytical accuracy, we first excluded 2230 participants lacking key data (missing data related to digital isolation) and further excluded 26 participants missing baseline sleep disorder data. This resulted in 5989 participants included in the cross-sectional analysis of the discovery sample. To examine the longitudinal association between digital isolation and sleep disorders, we excluded 3711 participants with confirmed sleep disorders at baseline. As a result, 2278 participants were included in the longitudinal analysis of the discovery sample.

To validate our findings, we used an independent validation sample comprising 4182 participants newly recruited in Wave 5 (2015) of NHATS. For these participants, data from Wave 5 to Wave 12 (2015‐2022) were used, encompassing seven waves of follow-up. In the validation sample, 723 participants lacking digital isolation-related information and 16 with missing baseline sleep disorder data were excluded. Thus, 3443 participants remained for the cross-sectional analysis in the validation sample. Similarly, for longitudinal analysis, 2197 participants with baseline sleep disorders were excluded, resulting in 1246 participants in the longitudinal validation cohort.

In the final analysis, the discovery and validation samples were combined, yielding a total of 9432 participants for the cross-sectional study. After excluding baseline cases of sleep disorders, 3524 participants were included in the longitudinal cohort. Combining the discovery and validation samples enhanced the reliability and generalizability of the findings.

### Definition of Digital Isolation

In this study, digital isolation was defined based on participants’ engagement across four key digital domains: (1) mobile phone ownership and usage (no ownership classified as digitally isolated in this domain), (2) functioning computer ownership and usage (no ownership or inability to use classified as digitally isolated), (3) electronic communication via email or text message in the past month (no engagement classified as isolated), and (4) engagement in other internet-based activities (excluding email or text message) in the past month (no participation classified as isolated). A score of 1 was assigned for nonengagement in each domain and 0 for engagement. These scores were summed to create a composite digital isolation score, ranging from 0 to 4, with higher scores indicating greater digital isolation. It is important to acknowledge that this scoring method treats all digital domains with equal weight and was not formally validated within this study. The distribution of the composite digital isolation scores in our sample ranged from 0 to 4. A substantial portion of participants scored at the extremes, reflecting either high digital engagement (0‐1) or high isolation (3-4). Based on this distribution, participants scoring ≤2 were categorized into the low isolation group, representing at least some engagement across 1 or 2 digital domains. Conversely, participants scoring ≥3 were categorized into the high isolation group, indicating a lack of engagement across the majority (3 or more) of the assessed domains. This cutoff aimed to distinguish between individuals with some level of digital connection and those experiencing a more substantial degree of digital disconnection. This classification formed the basis for subsequent statistical analyses [27].

### Sleep Disorders

This study aimed to assess sleep disorders based on participants’ sleep patterns during the past month. A total of 3 key indicators were used to identify potential sleep disorders. First, participants were asked whether they frequently took more than 30 minutes to fall asleep. Those who reported this occurring every night, most nights, or some nights were classified as having difficulty initiating sleep. Second, participants were asked whether they had difficulty returning to sleep after nocturnal awakenings. Those who experienced this every night, most nights, or sometimes were considered to have difficulty maintaining sleep. Finally, the study assessed whether respondents needed to use medication to help them sleep. Those who reported taking medication every night, almost every night, or on some nights were also classified as having a sleep disorder.

Based on these 3 questions, respondents were considered to have a sleep disorder if they experienced issues in any of the three areas and were assigned a value of 1 in the composite sleep disorder variable. If no issues were reported in these areas, they were assigned a value of 0. For missing or refused responses, the data were marked as missing values. This sleep disorder variable provided crucial data for subsequent statistical analyses, helping to reveal potential associations between sleep issues and other health variables [27].

### Covariates

We controlled for several covariates to minimize potential confounding. Respondents’ age was divided into two categories: 75 years and younger and 75 years and older, to analyze the effect of different age groups on the study results. Sex was based on respondents’ self-reports and categorized as male or female [28-30]. Marital status was classified as married or other statuses (such as cohabitating, separated, divorced, widowed, or never married) to evaluate the potential impact of marital status on the study variables [31-33]. Race and ethnicity included categories for non-Hispanic White, non-Hispanic Black, Hispanic, and other races (such as Native American, Asian, and Native Hawaiian) [34,35]. Smoking status was determined based on whether respondents were regular smokers, aiming to assess the impact of smoking on health outcomes [36].

Hearing impairment was defined as the use of hearing aids or reported difficulty hearing in quiet or noisy environments [37]. Vision impairment was determined based on respondents’ reports of difficulty seeing, even with corrective lenses, such as glasses [38]. For mental health, the Patient Health Questionnaire-2 scale was used to measure depressive symptoms. The total score ranged from 0 to 6, and participants with a score of 3 or higher were classified as having potential depression. Similarly, the Generalized Anxiety Disorder-2 scale assessed anxiety symptoms, with total scores ranging from 0 to 6, and a score of 3 or higher indicating potential anxiety [39-41]. In addition, we assessed respondents’ chronic conditions based on the self-reported number of chronic diseases and categorized them into groups with 3 or fewer conditions and more than 3 conditions [42]. These covariates were included in subsequent multivariable analyses to control for potential confounding effects of health and demographic characteristics on study outcomes.

### Statistical Analysis

All statistical analyses were sequentially conducted on the discovery, validation, and combined samples to ensure the robustness and generalizability of the findings. Descriptive analyses of baseline characteristics were first performed. *χ*^2^ tests were applied to examine differences among groups in baseline characteristics across levels of digital isolation (discovery vs validation samples). These descriptive analyses provided an initial overview of population characteristics and informed subsequent regression analyses.

In the cross-sectional analysis, multivariable logistic regression models were used to evaluate the association between digital isolation and sleep disorders. Model 1 was unadjusted, reflecting the crude association, whereas Model 2 adjusted for potential confounders to minimize bias. Covariates included in the adjusted model were age group, sex, race and ethnicity, smoking status, marital status, chronic disease status, hearing impairment, vision impairment, depression, and anxiety. Results were reported as odds ratios (ORs) with 95% CIs; *P* values <.05 were considered statistically significant. Analyses were initially conducted in the discovery sample to identify preliminary associations, then replicated in the validation sample to confirm robustness and consistency. Finally, analyses using the combined sample were performed to enhance statistical power and generate more generalizable conclusions.

For longitudinal analysis, Cox proportional hazards regression models were used to examine the link between digital isolation and the likelihood of incident sleep disorders among participants without baseline sleep disorders. Model 1 and Model 2 would also be calculated. Kaplan–Meier survival curves were used to estimate the incidence rate of sleep disorders across digital isolation groups, with differences assessed using the log-rank test. Survival analyses were initially performed in the discovery sample, validated in the validation sample, and subsequently applied to the combined sample to ensure generalizability.

In addition, subgroup analyses were conducted to explore potential effect modifications of digital isolation on specific populations (eg, different age groups, sex, and races and ethnicities). Interaction terms were added to the Cox regression model to detect potential interactions between subgroups, with corresponding *P* values used to assess significance. To ensure robustness, sensitivity analyses were also performed to verify the stability and consistency.

To assess the robustness of our longitudinal findings, particularly the nonsignificant result in the multivariable model of the validation sample, we planned a sensitivity analysis. This analysis involved incrementally introducing potential confounding variables (age group, sex, race and ethnicity, smoking status, marital status, hearing impairment, visual impairment, depression, anxiety, and chronic disease) into the Cox regression models to observe their impact on the hazard ratio between digital isolation and sleep disorders.

All statistics were performed using R (version 4.4.1; R Foundation for Statistical Computing), using 2-tailed tests with statistical significance set at *P*<.05.

### Ethical Considerations

The study protocol was approved by the Ethics Committee of Union Hospital, Tongji Medical College, Huazhong University of Science and Technology. The data for this study were obtained from the NHATS study, which is a publicly available dataset. The original NHATS study was approved by the Institutional Review Board of Johns Hopkins University, and all participants provided written informed consent before data collection. The dataset provided for public use is fully deidentified to ensure participant privacy and confidentiality.

## Results

### Study Population

**Figure 1.**
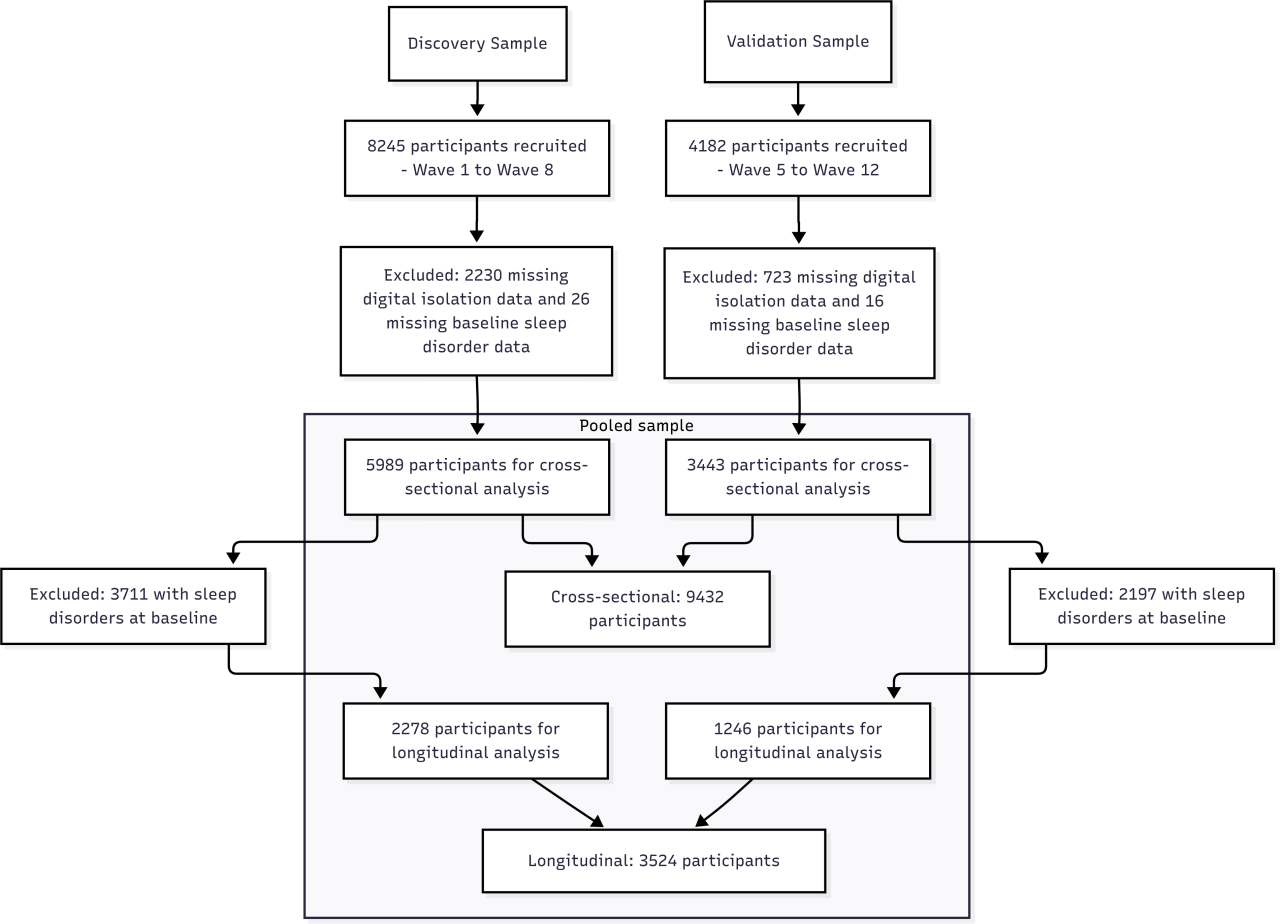
Study flowchart: derivation and selection of the final analytical sample.

Figure 1 shows a flowchart illustrating participant inclusion. Table 1 presents the baseline characteristics of the study population. In the discovery sample (n=5989), 54.73% (3279/5989) of participants were aged 75 years and older, whereas in the validation sample (n=3443), this proportion was slightly lower at 48.57% (1673/3443). In the combined sample (n=9432), 52.5% (4952/9432) of participants were aged 75 years and older. Regarding sex distribution, females comprised 55.82% (3343/5989) of the discovery sample and 55.27% (1903/3443) of the validation sample, resulting in an overall female proportion of 55.62% (5246/9432) in the combined sample. Analysis of marital status revealed that approximately 52.81% (4981/9432) of participants were married across the combined sample. In terms of racial and ethnic composition, non-Hispanic Whites were the dominant group (6503/9432, 68.95%), while non-Hispanic Blacks and Hispanics accounted for 20.48% (1932/9432) and 5.59% (527/9432), respectively. In addition, the distribution of digital isolation differed significantly between samples; the proportion of the high-isolation group was lower in the validation sample compared to the discovery sample (960/3443, 27.88% vs 2166/5989, 36.17%).

**Table 1.** Baseline characteristics of the study population. Baseline characteristics were compared between the discovery and validation samples. Categorical variables were analyzed using *χ*^2^ tests.^a^

Variables	Discovery sample (n=5989)	Validation sample (n=3443)	Pooled sample (n=9432)
Age group (years), n (%)^b^			
<75	2710 (45.27)	1770 (51.43)	4480 (47.5)
≥75	3279 (54.73)	1673 (48.57)	4952 (52.5)
Sex, n (**%**)			
Female	3343 (55.82)	1903 (55.27)	5246 (55.62)
Male	2646 (44.18)	1540 (44.73)	4186 (44.38)
Marital status, n (%)^c^			
Married	3195 (53.35)	1786 (51.87)	4981 (52.81)
Others	2788 (46.55)	1653 (48.01)	4441 (47.08)
Race and ethnicity, n (**%**)			
White and non-Hispanic	4204 (70.2)	2299 (66.77)	6503 (68.95)
Black and non-Hispanic	1249 (20.85)	683 (19.84)	1932 (20.48)
Hispanic	313 (5.23)	214 (6.22)	527 (5.59)
Other^d^	159 (2.65)	114 (3.31)	273 (2.89)
Smoking status, n (%)			
Nonsmoker	2828 (47.22)	1713 (49.75)	4541 (48.14)
Smoker	3158 (52.73)	1725 (50.10)	4883 (51.77)
Hearing impairment, n (**%**)			
No	4654 (77.71)	2713 (78.8)	7367 (78.11)
Yes	1313 (21.92)	713 (20.71)	2026 (21.48)
Visual impairment, n (**%**)			
No	3619 (60.43)	2006 (58.26)	5625 (59.64)
Yes	2352 (39.27)	1416 (41.13)	3768 (39.95)
Depression, n (%)^b^			
No	5146 (85.92)	3020 (87.71)	8166 (86.58)
Yes	843 (14.08)	423 (12.29)	1266 (13.42)
Anxiety, n (%)^c^			
No	5274 (88.06)	3083 (89.54)	8357 (88.6)
Yes	715 (11.94)	360 (10.46)	1075 (11.4)
Chronic disease, n (**%**)^e^			
≤3 diseases	4556 (76.07)	2659 (77.23)	7215 (76.49)
>3 diseases	1433 (23.93)	784 (22.77)	2217 (23.51)
Isolation group, n (%)^c^			
Low isolation	3823 (63.83)	2483 (72.12)	6306 (66.86)
High isolation	2166 (36.17)	960 (27.88)	3126 (33.14)
Items of digital isolation, n (**%**)			
Phone isolation	635 (10.6)	214 (6.22)	849 (9)
Computer isolation^c^	1925 (32.14)	1064 (30.9)	2989 (31.69)
Email isolation^c^	3600 (60.11)	1585 (46.04)	5185 (54.97)
Internet-based activity isolation^c^	3462 (57.81)	1609 (46.73)	5071 (53.76)

a Missing data were handled as follows: in the discovery sample, missing values were present for race and ethnicity (n=51), smoking status (n=3), marital status (n=5), dementia (n=1), vision impairment (n=12), and sleep disorders (n=10). In the validation sample, missing values were observed for race and ethnicity (n=107), smoking status (n=2), marital status (n=4), dementia (n=2), vision impairment (n=13), and sleep disorders (n=5). These missing data were excluded from the analysis.

b Statistically significant (*P*<.05).

c Statistically significant (*P*<.001).

d The "Other" group includes African Americans, Native Americans, Alaska Natives, Asians, Native Hawaiians, Pacific Islanders, and other unspecified races or ethnicities.

e The assessment of chronic disease burden is based on the cumulative number of the following conditions: heart disease, hypertension, arthritis, osteoporosis, diabetes, lung disease, stroke, cancer, and other illnesses.

### Cross-Sectional Association Between Digital Isolation and Sleep Disorders

Table 2 shows the cross-sectional association between digital isolation and sleep disorders. In the discovery sample, the prevalence of sleep disorders was significantly higher in the high-isolation group compared to the low-isolation group (67.03% vs 59.06%). In the unadjusted model (Model 1), the odds of sleep disorders were 41% higher in the high-isolation group compared to the low-isolation group (OR 1.41, 95% CI 1.26-1.57; *P*<.001). After adjusting for potential confounders, the association was still significant (OR 1.23, 95% CI 1.09-1.39; *P*<.001). Similar results were observed in the validation sample, with significantly increased odds of sleep disorders in the high-isolation group (OR 1.22, 95% CI 1.02‐1.47; *P*=.03), further supporting this association. In the combined sample, the adjusted odds of sleep disorders were 21% higher in the high-isolation group compared to the low-isolation group (OR 1.21, 95% CI 1.10‐1.34; *P*<.001), demonstrating a consistent cross-sectional relationship across the entire study population. The unadjusted models in all samples showed even stronger associations, suggesting a partial attenuation of the effect by the included covariates.

**Table 2.** Cross-sectional association between digital isolation and the risk of sleep disorder. This table presents the cross-sectional association between digital isolation and the risk of sleep disorder across discovery, validation, and pooled samples. Model 1 provides unadjusted odds ratios, while Model 2 adjusts for various covariates to control for confounding factors.

Sample or variables	Event, n/N (%)	Model 1, OR^a^ (95% CI)^b^	*P* value	Model 2, OR (95% CI)^c^	*P* value
Discovery sample					
Low isolation (Ref)^d^	2259/3823 (59.06)	1 (Ref)	—^e^	1 (Ref)	—
High isolation	1452/2166 (67.03)	1.41 (1.26-1.57)	<.001	1.23 (1.09-1.39)	.001
Validation sample					
Low isolation (Ref)	1524/2483 (61.38)	1 (Ref)	—	1 (Ref)	—
High isolation	673/960 (70.1)	1.48 (1.26-1.73)	<.001	1.22 (1.02-1.47)	.03
Pooled sample					
Low isolation (Ref)	3783/6306 (59.98)	1 (Ref)	—	1 (Ref)	—
High isolation	2125/3126 (67.96)	1.42 (1.29-1.55)	<.001	1.21 (1.10-1.34)	<.001

a OR: odds ratio.

b Model 1: unadjusted logistic regression model.

c Model 2: logistic regression model adjusted for age group, sex, race and ethnicity (white and non-Hispanic), smoking status, marital status, chronic disease (binary), hearing impairment, visual impairment, depression, and anxiety.

d Ref: reference category.

e Not applicable.

### Longitudinal Association Between Digital Isolation and Sleep Disorders

Table 3 summarizes the longitudinal association between digital isolation and incident sleep disorders among participants without baseline sleep disorders. In the discovery sample, the unadjusted Cox regression analysis showed a significantly increased hazard of sleep disorders in the high-isolation group (hazard ratio [HR] 1.30, 95% CI 1.15‐1.47; *P*<.001). After adjusting for age, sex, race and ethnicity, and other confounders, the association remained significant (HR 1.21, 95% CI 1.05‐1.38; *P*=.006). In the validation sample, the unadjusted model also showed a higher hazard in the high-isolation group (HR 1.29, 95% CI 1.07‐1.55; *P*=.008), but this association was no longer statistically significant after adjustment (HR 1.11, 95% CI 0.91‐1.36; *P*=.30), indicating a less clear longitudinal relationship in this specific sample. In the combined sample, the adjusted hazard of sleep disorders in the high-isolation group was 17% higher (HR 1.17, 95% CI 1.05‐1.31; *P*=.005), providing overall support for a longitudinal association, although the nonsignificant finding in the validation sample warrants further investigation. The unadjusted HRs were higher across all samples, suggesting that the included covariates partially explain the association. To ensure the validity of our Cox proportional hazards models, we assessed the proportional hazards assumption for all covariates using the Schoenfeld residuals test in R. The global test results indicated that the proportional hazards assumption was not significantly violated in any of the samples: discovery sample (*χ*²_12_=5.71; *P*=.93), validation sample (*χ*²_11_=4.49; *P*=.95), and pooled sample (*χ*²_12_=4.94; *P*=.96). Furthermore, the individual covariate tests also showed no significant violations of the proportional hazards assumption (all *P*>.05). The detailed results of the test are presented in the supplementary material (see Table S1 in Multimedia Appendix 1).

**Table 3.** Association between digital isolation and the risk of developing sleep disorders in baseline sleep disorder–free samples using Cox regression analysis.

Sample or variables	Event, n/N (%)	Model 1, HR^a^ (95% CI)^b^	*P* value	Model 2, HR (95% CI)^c^	*P* value
Discovery sample					
Low isolation (Ref)^d^	762/1564 (48.73)	1 (Ref)	—^e^	1 (Ref)	—
High isolation	385/714 (53.92)	1.30 (1.15-1.47)	<.001	1.21 (1.05-1.38)	.006
Validation sample					
Low isolation (Ref)	536/959 (55.88)	1 (Ref)	—	1 (Ref)	—
High isolation	140/287 (48.78)	1.29 (1.07-1.55)	.008	1.11 (0.91-1.36)	.30
Pooled sample					
Low isolation (Ref)	1298/2523 (51.45)	1 (Ref)	—	1 (Ref)	—
High isolation	525/1001 (52.45)	1.31 (1.18-1.45)	<.001	1.17 (1.05-1.31)	.005

a HR: hazard ratio.

b Model 1: unadjusted Cox proportional hazards model.

c Model 2: Cox proportional hazards model adjusted for age group, sex, race and ethnicity, smoking status, marital status, hearing impairment, visual impairment, depression, anxiety, and chronic disease (binary).

d Ref: reference category.

e Not applicable.

### Association Between Digital Isolation Components and Sleep Disorders

Table 4 displays the link between different items of digital isolation and sleep disorders. In the discovery sample, phone isolation was not significantly associated with sleep disorders (HR 1.01; *P*=.88), but in the validation sample, phone isolation significantly increased the hazard of sleep disorders (HR 1.46; *P*=.03). Computer isolation, email isolation, and internet-based activity isolation were all significantly associated with sleep disorders in both the discovery and combined samples, with adjusted HRs ranging from 1.16 to 1.20 (*P*<.05). However, in the validation sample, the associations for computer isolation and internet-based activity isolation lost significance after adjustment. These findings suggest that different aspects of digital isolation may have varying impacts on sleep disorder risk, and these associations may differ between the discovery and validation samples.

**Table 4.** Association between digital isolation items and the risk of developing sleep disorders.

Sample or variables	Model 1, HR^a^ (95% CI)^b^	*P* value	Model 2, HR (95% CI)^c^	*P* value
Discovery sample				
Phone isolation	1.07 (0.89-1.29)	.46	1.01 (0.84-1.23)	.88
Computer isolation	1.27 (1.12-1.44)	<.001	1.20 (1.04-1.38)	.01
Email isolation	1.28 (1.14-1.44)	<.001	1.18 (1.04-1.34)	.01
Internet-based activity isolation	1.26 (1.13-1.42)	<.001	1.16 (1.02-1.32)	.02
Validation sample				
Phone isolation	1.65 (1.19-2.28)	.003	1.46 (1.04-2.07)	.03
Computer isolation	1.20 (1.01-1.44)	.046	1.11 (0.91-1.34)	.29
Email isolation	1.41 (1.21-1.65)	<.001	1.21 (1.01-1.44)	.03
Internet-based activity isolation	1.30 (1.11-1.52)	.001	1.11 (0.94-1.32)	.22
Pooled sample				
Phone isolation	1.18 (1.01-1.39)	.04	1.09 (0.93-1.29)	.29
Computer isolation	1.25 (1.13-1.39)	<.001	1.16 (1.04-1.30)	.008
Email isolation	1.33 (1.21-1.46)	<.001	1.19 (1.08-1.32)	.001
Internet-based activity isolation	1.28 (1.17-1.41)	<.001	1.15 (1.04-1.27)	.008

a HR: hazard ratio.

b Model 1: unadjusted Cox proportional hazards model.

c Model 2: Cox proportional hazards model adjusted for age group, sex, marital status, race, smoking status, hearing impairment, visual impairment, depression, anxiety, and chronic disease.

### Kaplan–Meier Survival Curve and Forest Plot Analysis

**Figure 2.**
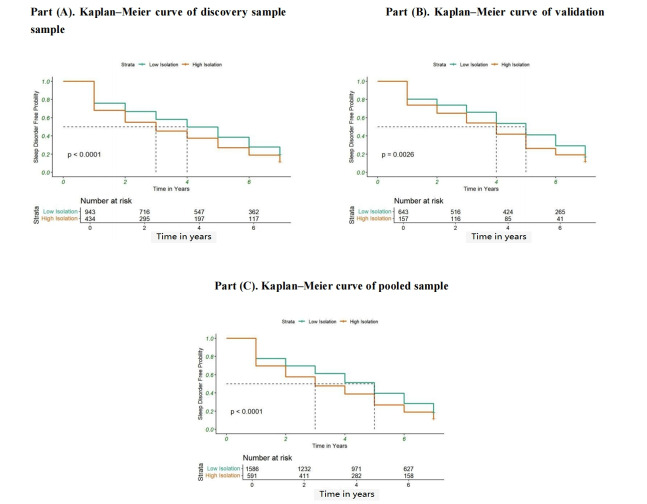
Kaplan–Meier curves of the relationship between digital isolation and developing sleeping disorder.

**Figure 3.**
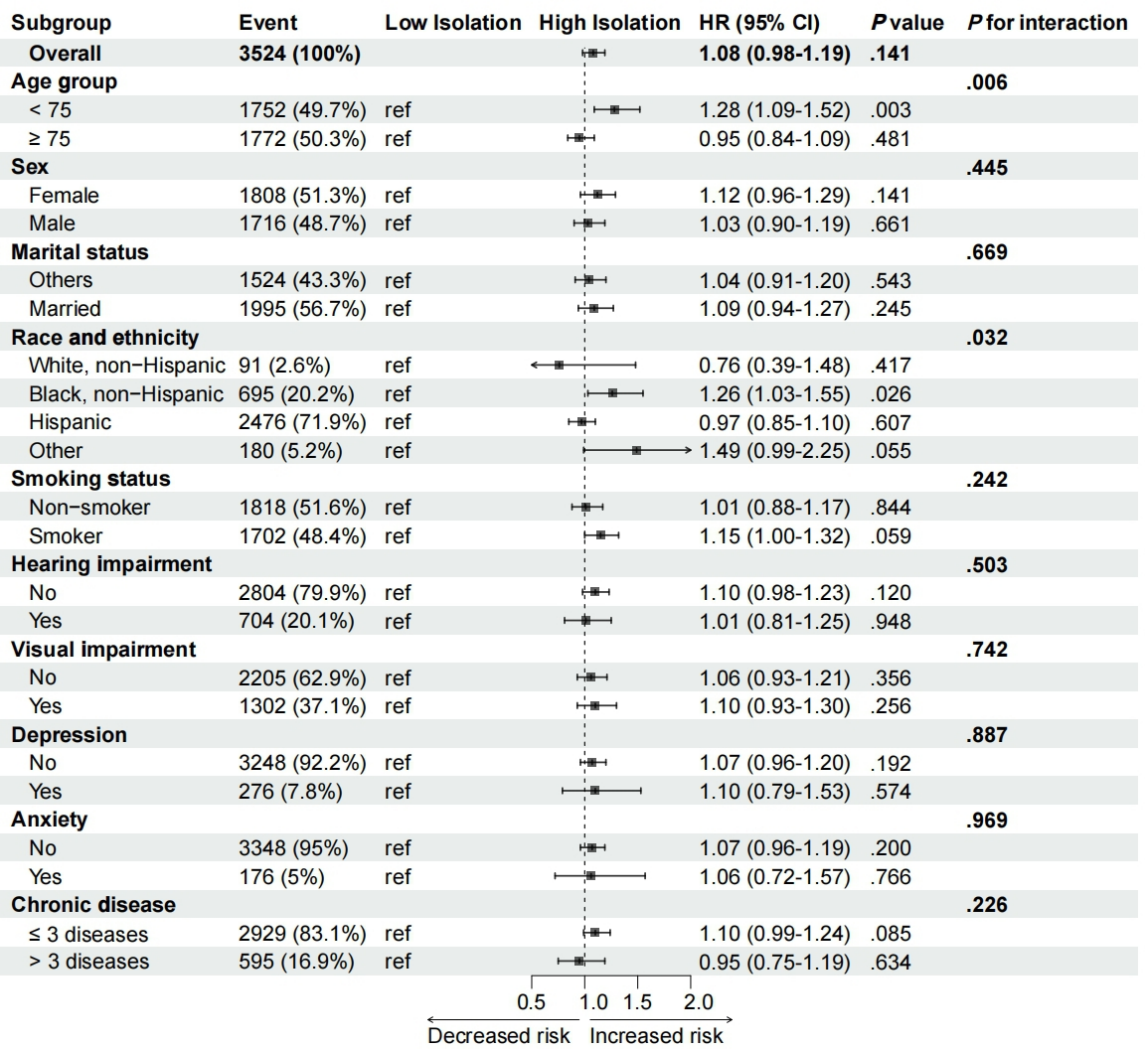
Subgroup analysis of digital isolation’s impact on sleep disorder risk across various demographic and health factors. HR: hazard ratio; ref: reference.

Kaplan–Meier survival curves further illustrated the incidence of sleep disorders during follow-up across different digital isolation groups (see Figure 2). The incidence of sleep disorders was significantly higher in the high-isolation group compared to the low-isolation group, with the log-rank test showing a statistically significant difference between the two groups (*P*<.001). In addition, the forest plot in Figure 3 demonstrated the link between digital isolation and sleep disorder among subgroups. The analysis showed consistent effects of digital isolation across different subgroups (eg, age, sex, and race and ethnicity), but stronger effects were observed in certain subgroups, such as the older age group and female participants, where interaction term *P* values indicated significant heterogeneity (*P*<.05).

### Sensitivity Analysis

Stepwise sensitivity analyses were conducted in the validation sample to examine how the association between digital isolation and incident sleep disorders was attenuated (see Table S2 in Multimedia Appendix 1). In the unadjusted model (Model 1), digital isolation was significantly associated with an increased risk of sleep disorders (HR 1.29; *P*=.008). After sequential adjustment for demographic characteristics (sex, marital status, and race), smoking status, hearing impairment, and visual impairment (Models 2‐5), the HR for digital isolation was attenuated but remained marginally significant (eg, Model 5: HR 1.23; *P*=.04). A key change emerged after adjusting for mental health factors. With the inclusion of depression (Model 6), the association between digital isolation and sleep disorders became non-significant (HR 1.20; *P*=.08), while depression emerged as a strong predictor (HR 1.52; *P*=.005). Adding anxiety (Model 7) revealed it as another significant predictor (HR 1.65; *P*=.01), while digital isolation remained nonsignificant (HR 1.18; *P*=.11). In the fully adjusted model (Model 9), which also included chronic disease burden and age group, digital isolation was no longer significantly associated with sleep disorders (HR 1.11; *P*=.30). In this model, depression (HR 1.52; *P*=.01), anxiety (HR 1.67; *P*=.009), chronic disease burden (HR 1.34; *P*=.006), and older age (HR 1.32; *P*=.001) were all independent and significant predictors. These analyses suggest that the initial association between digital isolation and incident sleep disorders was largely explained by confounding or mediation from mental health status, chronic disease burden, and age.

## Discussion

This study explored the association between digital isolation and sleep disorders using both cross-sectional and longitudinal designs. The results indicated a significant association between digital isolation and sleep disorders in both univariate and multivariate analyses within the discovery and combined samples. However, it is important to note that the hazard ratios for these longitudinal associations were modest (HR 1.21 and HR 1.17, respectively). However, in the validation sample, while a significant association was observed in the univariate analysis, the effect of digital isolation weakened as confounding factors were introduced, ultimately losing significance in the multivariate model. This suggests that health conditions such as depression, anxiety, and chronic diseases may play a role in the association between digital isolation and sleep disorders, potentially acting as mediating factors.

The findings align with previous literature, particularly regarding the significant association between digital isolation and sleep disorders. For example, earlier studies have shown that social isolation, depression, and anxiety are closely related to sleep disorders [43-45]. Our study’s univariate findings align with these previous studies, indicating a significant association between digital isolation and sleep disorders before adjusting for other confounders. However, as we incrementally introduced confounders, particularly health conditions, the effect of digital isolation diminished or disappeared. This is consistent with some studies suggesting that mental health factors such as depression and anxiety may act as key mediators between digital isolation and sleep disorders [46]. In contrast to our findings of a reduced effect, some studies have emphasized the independent effects of digital isolation; this discrepancy may be due to differences in study design, sample characteristics, or data handling [24,47].

In the validation sample, we observed that digital isolation was significantly associated with sleep disorders in the univariate model, but this association lost significance in the multivariate analysis. Conversely, in the discovery and combined samples, a significant association between digital isolation and sleep disorders persisted in both univariate and multivariate models, although the adjusted hazard ratios suggest a modest increase in risk. This discrepancy suggests that the independent effect of digital isolation in the validation sample diminished as more confounders were introduced, possibly due to differences in the health characteristics of individuals in the validation sample. The prevalence or severity of depression, anxiety, and chronic diseases in the validation sample may differ significantly from those in the discovery and combined samples, leading to stronger confounding effects. In addition, differences in the representativeness and statistical power of the validation sample could also explain why the multivariate model was not significant in this sample. Therefore, further research should investigate the mediating role of these health factors in different populations, especially in the validation sample.

The results of the multivariate model indicate that the association between digital isolation and sleep disorders might be influenced by depression, anxiety, and chronic diseases, possibly through mediating pathways. Mental health factors such as depression and anxiety have been confirmed in numerous studies as important contributors to sleep disorders [48-50]. In our study, the observed reduction in the effect of digital isolation upon the introduction of these mental health factors suggests a potential mediating role for psychological health problems in this relationship. Similarly, chronic diseases showed a significant association with sleep disorders in the multivariate model, suggesting a potential direct and indirect influence on sleep quality by increasing psychological stress or exacerbating depression and anxiety, thereby influencing the occurrence of sleep disorders.

One of the major strengths of this study is the combination of cross-sectional and longitudinal research designs. This approach allowed us not only to analyze the association between digital isolation and sleep disorders at a specific time point but also to explore dynamic changes over time through longitudinal data, which enhances the ability to infer causality. Furthermore, the study systematically evaluated the impact of each confounder by incrementally introducing them through sensitivity analysis, strengthening the interpretability and robustness of the results. The large sample size and independent analyses in both the discovery and validation samples provided opportunities to verify the robustness of the findings. The combined sample ensured the generalizability and applicability of the results.

Despite these strengths, there are some limitations to this study. First, the data were self-reported, particularly for psychological health variables such as depression and anxiety, which may be influenced by subjective factors, leading to bias. Future studies should use more objective health assessment tools to ensure data accuracy. Second, although we controlled for a variety of confounders through multivariate analysis, unmeasured or unaccounted-for potential confounders may still exist, potentially influencing the association between digital isolation and sleep disorders. Future research could introduce more relevant factors for a more comprehensive analysis. Third, our composite measure of digital isolation treated all digital domains equally, and the selection of cutoffs (≤2 vs ≥3) was based on the distribution of our data rather than external validation. Finally, while significant results were obtained in the discovery and combined samples, the nonsignificant findings in the multivariate model of the validation sample suggest that sample characteristic differences may influence the results. Future studies should further explore the potential reasons for these differences.

Future research should use longitudinal designs with longer follow-up periods to further investigate the complex interactions between digital isolation and other health outcomes, such as depression and anxiety, specifically examining the directionality and temporal relationships in different age groups and cultural contexts. In addition, future longitudinal studies could incorporate formal mediation analyses to further reveal the long-term impact of digital isolation on sleep outcomes, particularly the mediating role that specific psychological health problems (eg, severity of depressive symptoms and specific anxiety subtypes) may play in this process. Randomized controlled trials or other intervention studies could also be used to assess whether interventions aimed at reducing digital isolation (eg, digital literacy training and provision of accessible technology and support) help improve sleep quality, providing practical evidence for public health interventions. Furthermore, future research could explore potential moderators of this association, such as social support networks and preexisting health conditions.

This study revealed an association between digital isolation and sleep disorders through a combination of cross-sectional and longitudinal designs. Although digital isolation was significantly associated with sleep disorders before controlling for confounders, the association weakened and lost significance with the introduction of confounders such as depression, anxiety, and chronic diseases. This suggests that psychological health problems may mediate the link between digital isolation and sleep disorders. Therefore, following public health interventions need focus on improving mental health to mitigate the adverse impacts of the digital divide on sleep quality in aging populations.

## Supplementary material

10.2196/75328 Multimedia Appendix 1 Schoenfeld residuals test results, sensitivity analysis, and Kaplan–Meier survival curves.

## Abbreviations

HR: hazard ratio
NHATS: National Health and Aging Trends Study
OR: odds ratio
