# Supplementary material for: Association Between Digital Isolation and Sleep Disorders in Older Adults: Cross-Sectional and Longitudinal Study Using National Health and Aging Trends Study (NHATS) Data
Source: J Med Internet Res. 2025 Aug 6;27:e75328. doi: 10.2196/75328 (PMC12327964; doi:10.2196/75328)

Table S1. Schoenfeld residuals test results for Cox proportional hazards models

|  | Discovery Sample p-value | Validation Sample p-value | Pooled Sample p-value |
| --- | --- | --- | --- |
| age_group | 0.9113 | 0.5551 | 0.4053 |
| sex | 0.4571 | 0.8217 | 0.5334 |
| marital_status | 0.706 | 0.624 | 0.7202 |
| race | 0.4368 | 0.8809 | 0.565 |
| smoking_status | 0.2228 | 0.5744 | 0.7387 |
| hearing_impairment | 0.9269 | 0.9308 | 0.8946 |
| visual_impairment | 0.7451 | 0.2279 | 0.5408 |
| depression | 0.3916 | 0.2292 | 0.9098 |
| anxiety | 0.373 | 0.8731 | 0.3776 |
| chronic_disease | 0.7354 | 0.7469 | 0.6738 |
| isolation_group | 0.7889 | 0.8084 | 0.326 |

Table S2. Sensitivity analysis of the association between digital isolation and incident sleep disorders: stepwise covariate adjustment in the validation sample

| Model | Covariates included in the Model step by step | Digital isolation (High vs. Low) HR (95% CI) | P-value |
| --- | --- | --- | --- |
| 1 | Unadjusted model | 1.29 (1.07 - 1.55) | 0.008 |
| 2 | Model 1+sex+marital status+race | 1.24 (1.02 - 1.51) | 0.033 |
| 3 | Model 2+smoking status | 1.26 (1.03 - 1.54) | 0.023 |
| 4 | Model 3+hearing impairment | 1.24 (1.01 - 1.51) | 0.035 |
| 5 | Model 4+visual impairment | 1.23 (1.01 - 1.51) | 0.042 |
| 6 | Model 5+depression | 1.20 (0.98 - 1.46) | 0.082 |
| 7 | Model 6+anxiety | 1.18 (0.96 - 1.44) | 0.113 |
| 8 | Model 7+chronic disease | 1.17 (0.96 - 1.43) | 0.129 |
| 9 | Model 8+age agroup | 1.11 (0.91 - 1.36) | 0.298 |

Figure S1. Kaplan-Meier survival curves illustrating sleep disorder-free probability by digital isolation group in the discovery sample.


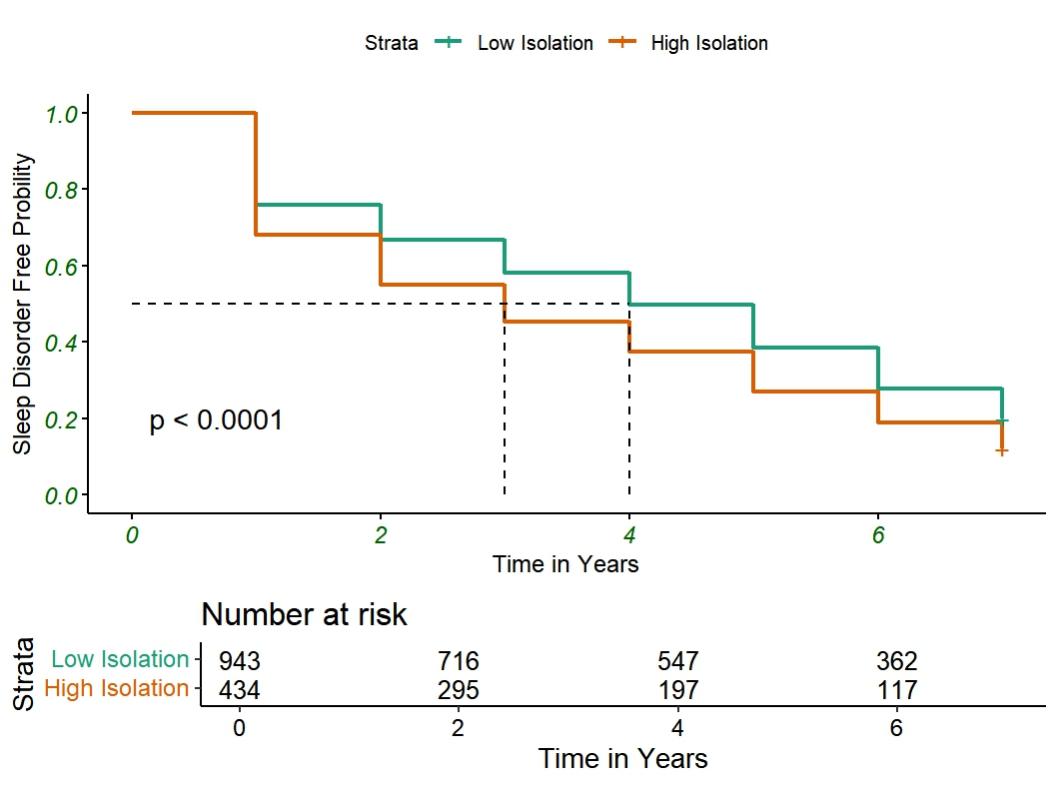


Figure S2. Kaplan-Meier survival curves depicting sleep disorder-free probability by digital isolation group in the validation sample.


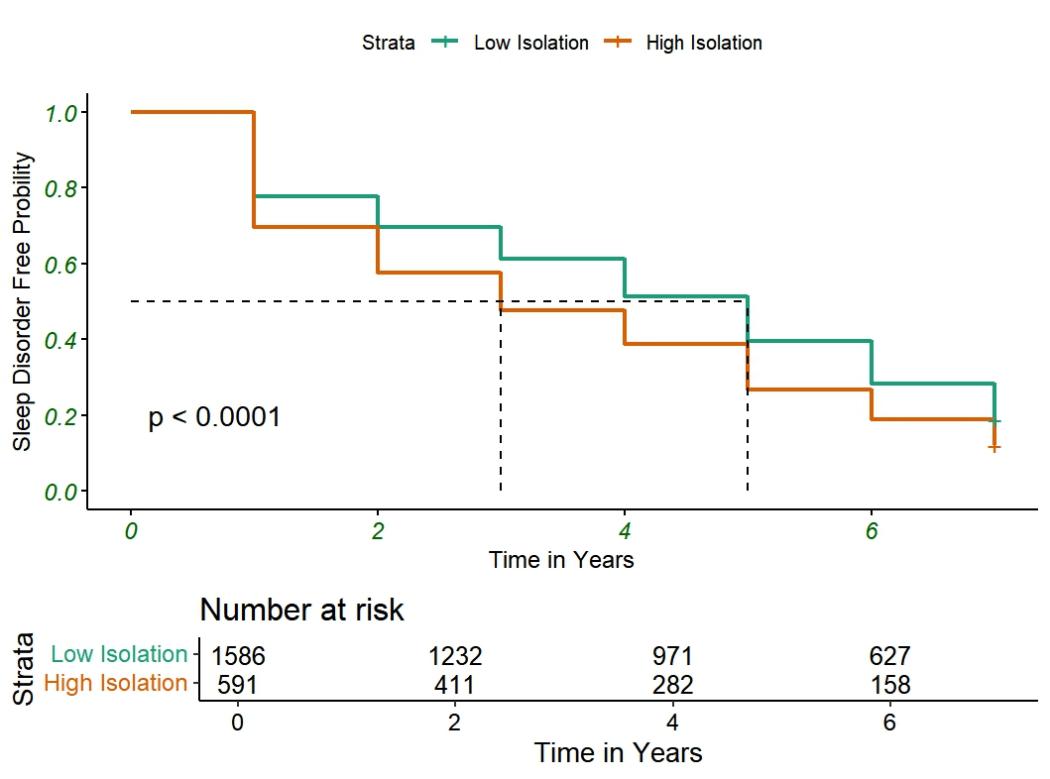


Figure S3. Kaplan-Meier survival curves showing sleep disorder-free probability by digital isolation group in the pooled sample.


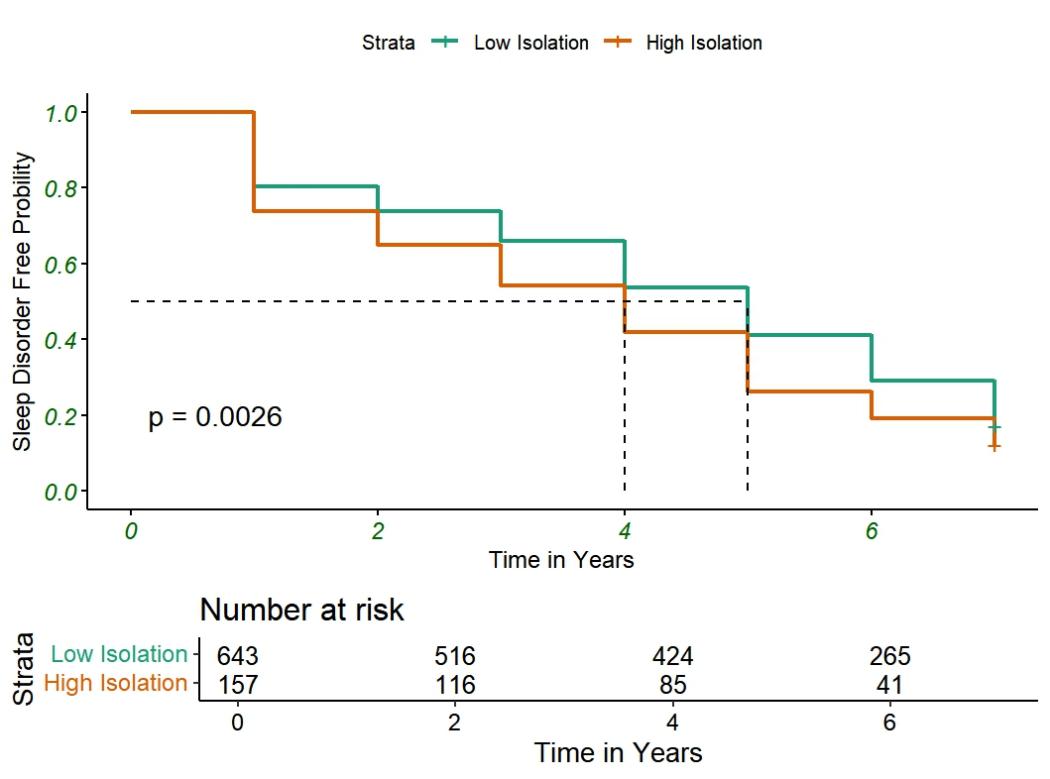

Supplement: Multimedia Appendix 1 [file jmir-v27-e75328-s001.docx]
